# Imprinted and ancient gene: a potential mediator of cancer cell survival during tryptophan deprivation

**DOI:** 10.1186/s12964-018-0301-7

**Published:** 2018-11-22

**Authors:** Petr Tomek, Shanti K. Gore, Chloe L. Potts, Cristin G. Print, Michael A. Black, Ariane Hallermayr, Michael Kilian, Evelyn Sattlegger, Lai-Ming Ching

**Affiliations:** 1 Auckland Cancer Society Research Centre, Faculty of Medical and Health Sciences, University of Auckland, Auckland, New Zealand; 2 Department of Molecular Medicine & Pathology, Faculty of Medical and Health Sciences, University of Auckland, Auckland, New Zealand; 3 Department of Biochemistry, University of Otago, Dunedin, New Zealand; 4 Institute of Natural and Mathematical Sciences, Massey University, Auckland, New Zealand; 5 Medical Genetics Center (MGZ), Munich, Germany; 6 German Cancer Research Center (DKFZ), Heidelberg, Germany

**Keywords:** IMPACT, IDO1, Immune suppression, Cancer cell survival

## Abstract

**Background:**

Depletion of tryptophan and the accumulation of tryptophan metabolites mediated by the immunosuppressive enzyme indoleamine 2,3-dioxygenase 1 (IDO1), trigger immune cells to undergo apoptosis. However, cancer cells in the same microenvironment appear not to be affected. Mechanisms whereby cancer cells resist accelerated tryptophan degradation are not completely understood. We hypothesize that cancer cells co-opt IMPACT (the product of *IMPrinted and AnCienT* gene), to withstand periods of tryptophan deficiency.

**Methods:**

A range of bioinformatic techniques including correlation and gene set variation analyses was applied to genomic datasets of cancer (The Cancer Genome Atlas) and normal (Genotype Tissue Expression Project) tissues to investigate *IMPACT*’s role in cancer. Survival of IMPACT-overexpressing GL261 glioma cells and their wild type counterparts cultured in low tryptophan media was assessed using fluorescence microscopy and MTT bio-reduction assay. Expression of the Integrated Stress Response proteins was measured using Western blotting.

**Results:**

We found *IMPACT* to be upregulated and frequently amplified in a broad range of clinical cancers relative to their non-malignant tissue counterparts. In a subset of clinical cancers, high *IMPACT* expression associated with decreased activity of pathways and genes involved in stress response and with increased activity of translational regulation such as the mTOR pathway. Experimental studies using the GL261 glioma line showed that cells engineered to overexpress IMPACT, gained a survival advantage over wild-type lines when cultured under limiting tryptophan concentrations. No significant difference in the expression of proteins in the Integrated Stress Response pathway was detected in tryptophan-deprived GL261 IMPACT-overexpressors compared to that in wild-type cells. IMPACT-overexpressing GL261 cells but not their wild-type counterparts, showed marked enlargement of their nuclei and cytoplasmic area when stressed by tryptophan deprivation.

**Conclusions:**

The bioinformatics data together with our laboratory studies, support the hypothesis that IMPACT mediates a protective mechanism allowing cancer cells to overcome microenvironmental stresses such as tryptophan deficiency.

**Electronic supplementary material:**

The online version of this article (10.1186/s12964-018-0301-7) contains supplementary material, which is available to authorized users.

## Background

Cancers utilise a diverse range of strategies to escape elimination by the patient’s immune system [1]. The tryptophan catabolising enzyme, indoleamine 2,3-dioxygenase 1 (IDO1) mediates one of the key immune suppressive mechanisms for a number of clinical malignancies [2–4]. Depletion of tryptophan by IDO1 and concomitant production of tryptophan metabolites such as kynurenine, induce immune T-lymphocytes to undergo apoptosis [5, 6], as well as promoting activation and differentiation of immunosuppressive regulatory T-cells [6, 7]. In contrast, cancer cells in the same microenvironment appear not to be affected by the accelerated tryptophan catabolism. Mechanisms whereby cancer cells overcome IDO1-mediated tryptophan deprivation are of intense interest and scientific speculation. HeLa cells overexpressing IDO1 in one study, were found to have increased expression of tryptophan transporters and up-regulation of genes involved with amino acid metabolism and cell survival controlled by the ATF4 stress response transcription factor [8]. Another pertinent study showed that skin fibroblasts express an abundance of a protein called IMPACT (product of the gene named *IMPrinted and AnCienT*) [9–11]. High expression of IMPACT in skin was suggested to render those cells more resistant to IDO1-mediated tryptophan deprivation [12]. The same study found that skin fibroblasts upregulate IMPACT when cultured in the tryptophan-free media [12], suggesting that IMPACT regulates an adaptive stress response that enables cells to survive periods of tryptophan deprivation. The role of IMPACT in cancer is very much understudied, but in normal mammalian cells, IMPACT has been shown to have a similar role as the YIH1 protein in yeast; inhibiting activation of the general control non-derepressible 2 (GCN2) kinase that senses amino acid scarcity [13, 14]. IMPACT is preferentially expressed in mouse brain tissue [11], and the abundance of IMPACT correlates inversely with levels of phosphorylated alpha-subunit of the eukaryotic translation initiation factor 2 (eIF2α) in different areas of the brain. A subsequent study showed that abundance of IMPACT increases during differentiation of neurons whilst GCN2 activation is lowered. Endogenous IMPACT was shown to promote neurite outgrowth, whereas GCN2 inhibited neuritogenesis [15]. These seminal studies establish an important role of the IMPACT/GCN2 nexus in the development of the nervous system. The authors suggested that IMPACT abundance ensures a constant high level of translation under conditions of amino acid starvation in specific neuronal cells, through inhibition of activation of the GCN2-dependent stress response pathway.

In the studies of Habibi and colleagues [12], IMPACT expression in T cells was found to be significantly lower to that of skin cells. The role of IMPACT/GCN2 nexus in IDO1/TDO dependent immune suppression remains controversial. On the basis that T cells with a targeted disruption of GCN2 are refractory to IDO1-induced anergy, Munn and co-workers suggested that GCN2 mediated proliferative arrest in response to IDO1 [16]. In contrast, the studies of Sonner et al. found no difference in the efficacy of GCN2-deficient and GCN2-proficient T cells against B16 melanomas [17]. In addition, there is increasing evidence of cell survival responses being regulated by IMPACT that are independent of GCN2. In that regard, IMPACT has been reported to control mammalian cell proliferation through its binding of the cell cycle regulatory protein CDK1 [18], as well as the cytoskeletal protein ACTIN [19]. Thus, there is accumulating evidence that IMPACT may play an important role in cell survival responses through its differential regulation of cell-type dependent stress response pathways.

There are very few studies of IMPACT in cancer. As part of our efforts to understand how cancer cells may survive low tryptophan concentrations when immune T cells in the same environment are induced to undergo apoptosis; we explored the association of IMPACT abundance and the resistance of the cancer cells to tryptophan scarcity. In this report, we present data from a bioinformatic meta-analysis that show *IMPACT* gene being abundantly expressed and frequently amplified in a broad range of human malignancies. In a subset of cancer types examined, high *IMPACT* expression was associated with low activity of stress response pathways and decreased expression of key stress response mRNAs. Conversely, increased IMPACT expression correlated with increased activity of pathways involved in translational regulation. GL261 glioma lines engineered to overexpress IMPACT were shown to remain viable to a greater extent than wild type GL261 cells when cultured under limiting tryptophan concentrations. Taken together, the data from the bioinformatics and the experimental studies reported here, suggest that high IMPACT expression benefits cancer cell survival during periods of accelerated tryptophan catabolism induced by IDO1.

## Methods

### Bioinformatics meta-analyses

Data manipulation, plotting and statistical analyses were performed in the R computing environment (v3.4.4). The figures were generated using *ggplot2* package (v2.2.1) [20]. RNA-sequencing data for cancer (TCGA; The Cancer Genome Atlas) and non-cancer (GTEx; Genotype-Tissue Expression project) samples were extracted from the RSEM expected_count dataset included in the UCSC Xena portal [21]. This dataset has been generated by Vivian and colleagues [22] by reprocessing the TCGA and GTEx RNA-sequencing data using the TOIL pipeline. While this reprocessing removed batch effects, a robust between-sample normalisation is still needed. Prior to normalisation, we removed non-protein-coding genes and genes not expressed in > 80% samples within all of the 52 tissue types analysed. Subsequently, we quantile normalised the dataset (19,446 genes) using *normalize.quantiles* function from the Bioconductor package *preprocessCore* (v1.32.0) in R. The conservative Quantile normalisation was selected as the most suitable normalisation procedure according to the comparison of several normalisation methods (Additional file 1).

Segmental copy number data and methylation data used in this study were measured at the TCGA genome characterisation centres using Affymetrix Genome-Wide Human SNP Array 6.0 platform and Illumina Infinium Human Methylation 450 platform, respectively. The data were downloaded from the UCSC Xena portal [23]. The segmental copy number data were converted to more instructive estimated copy number values by first computing their inverse log_2_ and then multiplying the resulting values by a factor of two. Methylation data (450 K) were processed as follows: Firstly, out of all 19 probes located at the transcription start site and the first exon of the human *IMPACT* gene, we excluded 2 probes that did not contain any values, and 4 probes that were consistently hyper-methylated (β-value ≥0.8) in the majority (≥ 75%) of the TCGA tumour samples. Subsequently, for each tumour sample, we calculated an arithmetic mean of β-values of the 13 remaining probes (cg22757447, cg13981356, cg24275769, cg03400437, cg03143886, cg24949251, cg18332806, cg02241481, cg13865352, cg19083143, cg25619607, cg03013329, cg03614916).

Gene sets studied were selected by performing an over-representation analysis of stress response genes examined in this study using databases GeneSetDB [24] and ConsensusPathDB [25]. From the initial 69 gene sets, the selection was narrowed down to nine minimally overlapping gene sets that were relevant to translational regulation and stress response. Preference was given to gene sets outside of the Gene Ontology terms to ensure non-redundancy and directionality. Relative activity of gene sets in each sample was calculated using Gene Set Variation Analysis (GSVA) [26]. This approach offers a considerable advantage over other gene set enrichment methods as the GSVA activity scores follow a close to normal distribution, permitting their use in the same way as gene expression values.

**Fig. 1:**
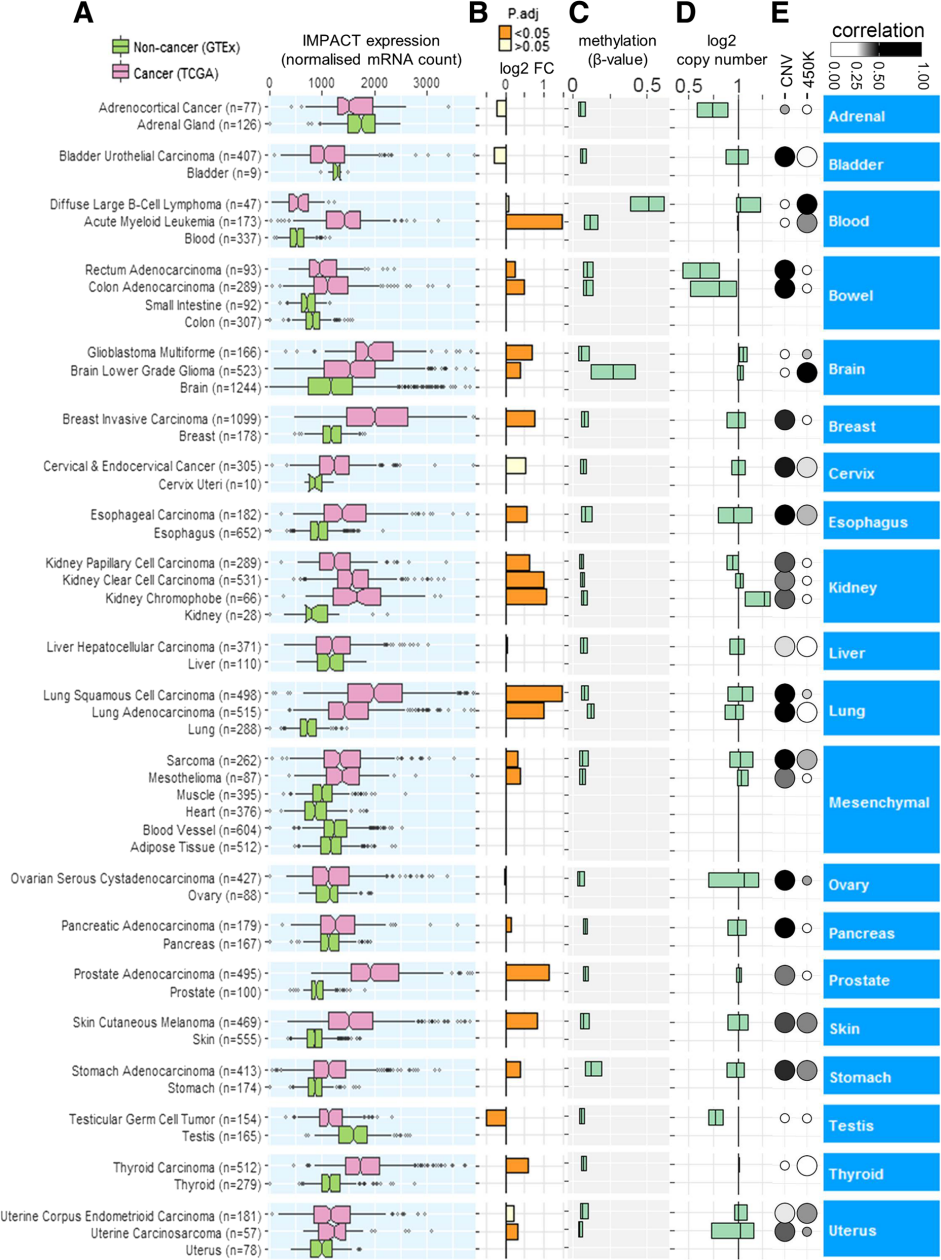
Amplification of *IMPACT* drives its increased expression in a wide range of human malignancies. **a)** *IMPACT* expression in tumours and non-cancer tissues. The middle line inside each box represents median, the left and right hinge denote lower and upper quartile, respectively, and whiskers extend to 1.5x interquartile range. Open circles denote outliers; and for clarity, 112 outliers have been removed. **b)** Differential expression of *IMPACT* between cancer and non-cancer tissues. Each bar indicates a fold change difference (log2 FC) between a log_2_ transformed median mRNA count in each cancer type and pooled non-cancer samples within each organ classification. **c)** Boxplot of methylation (mean β-value) of the *IMPACT* promoter site. β-values < 0.2 indicate hypo-methylation. **d)** Boxplot of the estimated copy number of *IMPACT*. The value of 1 represents a diploid copy number. The middle vertical line, and left and right hinges in the boxes in Fig. 1c and d indicate median, 25th and 75th percentile, respectively. **e**) Absolute values of Spearman’s rank correlations (grey scale gradient) between the expression of *IMPACT* and *IMPACT* copy number variation (CNV) or methylation of the *IMPACT* promoter (450 K). Larger circle area indicates statistically significant correlations (*P* ≤ 0.05) and vice versa. Tabulated values for Fig. 1e and additional details are available in Additional file 3

Statistical significance between the expression of *IMPACT* mRNA in malignant and non-malignant tissues (Fig. 1b) was estimated from 10^5^ Monte Carlo replications using function *permTS* (two-tailed) from the *perm* package (v1.0). Statistical significance of correlation values was estimated from 10^5^ permutations using the function *perm.cor.test* (two-tailed) from the *jmuOutlier* package (v1.3). *P* values were adjusted for multiple hypotheses testing using highly conservative Bonferroni algorithm.

### GL261 line and cell culture

The GL261 murine glioma cell line was obtained from the National Institute of Health, USA. The parental GL261 line and its daughter transfected lines generated for use in this study, were determined to be mycoplasma-free using PlasmoTest Mycoplasma Detection Kit (Invivogen, USA), and cultured at 37 °C and 5% CO_2_ in Dulbecco’s Modified Eagle Medium (DMEM) (Gibco, Grand Island, NY, USA), supplemented with 10% foetal calf serum (FCS) (Moregate, Bulimba, Australia), and antibiotics penicillin (100 U) and streptomycin (100 μg/mL) from Gibco.

### Generation of the GL261 line overexpressing IMPACT

cDNA encoding a full-length mouse IMPACT gene (Cat. No. MR204593; OriGene, Rockville, MD, USA) was cloned into the bicistronic mammalian expression vector F279-V5 [27] using Gateway technology. The correct insertion of the cDNA was verified by restriction enzyme digest and Sanger sequencing. The F279-V5 plasmid harbouring IMPACT cDNA was transfected into the cells using Lipofectamine LTX (Invitrogen, Carlsbad, USA), and the cells that integrated the plasmid into their genome were selected by culturing for at least 3 weeks in culture medium supplemented with the Puromycin antibiotic (Gibco). A polyclonal cell population that stably overexpressed IMPACT and showed doubling time and morphology comparable to that of the parental wild-type line was selected for all subsequent work.

### Western blotting of integrated stress response (ISR) proteins

GL261 cells (8 × 10^5^) were seeded in T-25 flasks in 7.5 mL DMEM F-12 media containing 50 μM tryptophan 12 h prior to the beginning of the experiment in order to limit the stress response induced by manipulation of the cells. The original medium was then substituted by a fresh medium containing either 50 μM or 5 μM tryptophan and the cells were incubated for a required time period prior to lysis. Whole cell lysates were separated on a precast Nu-page Bis-Tris gel (4–12%, 1.5 mm, Thermo Scientific, Rockford, IL, USA) under reducing and denaturing conditions in 1X MES SDS buffer (Novex, Carlsbad, CA, USA), and proteins transferred to a nitrocellulose membrane (0.45 μM pore size; BioRad, Germany) in ice-cold TRIS/Glycine buffer containing 20% methanol. After blocking the membrane with 5% milk in TBS/Tween20 buffer, the membrane was incubated with primary antibodies and subsequently with HRP conjugated IgGs of required reactivity at dilutions listed below. The membranes were incubated with the chemiluminescence substrate SuperSignal West Pico (Thermo Scientific) and imaged on a luminescent image analyser LAS-4000 (Fujifilm, Tokyo, Japan). After imaging, the membranes were stripped using Restore Western stripping buffer (Thermo Scientific) and re-probed for α-tubulin. Bands were quantified by integrating their pixel density using the Gel Analyzer module in ImageJ.

Antibodies used: IMPACT (1:500, NBP1–86221, Lot# R38453) is a rabbit pAb purchased from Novus Biologicals. eIF2α (1:1000, 5324S), p-eIF2α [Ser51] (1:1000, 3398S), ATF4 (1:1000, 11815S) and CHOP (1:500, 5554S) are rabbit mAbs from Cell Signalling Technology. α-tubulin (1:5000; T6074) was obtained from Sigma-Aldrich. Goat anti-rabbit IgG-HRP (1:5000, sc-2054, Lot# A3014) and goat anti-mouse IgG-HRP (1:5000, sc-2055) are products of Santa Cruz Biotechnology. IgG-HRP, α-tubulin and IMPACT antibodies were incubated with membranes in 5% milk in TBS/Tween20 buffer for 1 h at RT. eIF2α, p-eIF2α, ATF4 and CHOP antibodies were applied in 5% BSA overnight at 4 °C.

### Determination of cell viability and metabolic activity

Triplicate cell cultures were plated in a 96-well microplate at 4 × 10^3^ cells per well in 200 μL of tryptophan-free Dulbecco’s MEM F-12 medium (D9807–04; US Biological Life Sciences, Salem, MA, USA) supplemented with 2.2 g/L sodium bicarbonate (pH adjusted to 7.2), 5% (*v*/v) non-dialysed FCS and l-tryptophan as required. After five days of culture, the cells cultured in two separate microplates were processed for either fluorescence microscopy to determine viability or MTT assay [28] to assess metabolic activity:

i). Fluorescence microscopy

Each well received a viability indicator fluorescein diacetate (1 μM; Invitrogen, Eugene, OR, USA), an indicator of non-viable cells propidium iodide (3 μM; Sigma-Aldrich, St Louis, MO, USA), and the DNA dye Hoechst 33342 trihydrochloride (2 μM; Sigma-Aldrich) for the cell area determination. After 30 min of incubation at 37 °C, the medium containing the dyes was replaced with fresh medium and the cells were immediately imaged on the FLoid Cell Imaging Station (Life Technologies, Carlsbad, CA, USA) equipped with a 20X objective. Acquired 16-bit greyscale images were processed in ImageJ (v1.50e, courtesy of Wayne Rasband, National Institutes of Health, USA) as follows: The green colour gradient was applied to the fluorescein diacetate images and the image contrast was increased and normalised using *Enhance Contrast* function. The magenta hot colour gradient was applied to the propidium iodide images and the contrast and brightness was adjusted to reduce fluorescence signal not emitted from cell nuclei. To determine areas of the cells (Fig. 6b), nine central sites (45% well coverage) in each of the three wells per experimental condition were imaged on the high content microscope ImageXpress Micro XLS (Molecular Devices, Sunnyvale, CA, USA) using a 10x Plan Fluor objective. The Quad 5 Filter cube equipped with DAPI (λ_ex_ 390 nm) and FITC (λ_ex_ 485 nm) filters was employed to visualise Hoechst 33342 and fluorescein diacetate, respectively. Cells in the acquired images were segmented and their fluorescein diacetate stained areas were computed using an optimised Multi Wavelength Cell Scoring module in the MetaXpress high content analysis software (v 6.2.3, Molecular Devices).

ii). MTT assay

Each well received Thiazolyl Blue Tetrazolium Bromide (MTT; final concentration 500 μg.ml^− 1^; Sigma-Aldrich). When formazan crystals were observed in the wells (typically after 30 min incubation at 37 °C), the plate was centrifuged at 1600 *g* for 15 min, the supernatant was decanted and the formazan crystals were dissolved in DMSO prior to readout on the EnSpire 2300 plate reader (Perkin-Elmer, Singapore). The background absorbance at 690 nm was subtracted from the formazan absorbance at 570 nm to obtain a normalised absorbance value that is considered directly proportional to the metabolic activity of the cells.

## Results

### Expression of *IMPACT* in normal and tumour tissues

As a first step towards understanding the role of *IMPACT* in cancer, we carried out a bioinformatic meta-analysis of *IMPACT* mRNA expression in 28 cancer types from the TCGA compared to that in 24 corresponding normal tissues from the GTEx data set belonging to 20 organ classifications. The majority of the cancers examined expressed significantly (two-tailed permutation test, *P* ≤ 0.05) higher levels of *IMPACT* compared to their normal tissue counterparts (Fig. 1a). Acute myeloid leukaemia, pulmonary squamous cell carcinoma, prostatic adenocarcinoma and renal chromophobe tumours showed the highest expression of *IMPACT* relative to matching normal tissues (2.1-fold to 2.8-fold greater, respectively; Fig. 1b). Testicular germ cell tumour was the only cancer in the dataset with significantly lower *IMPACT* expression (1.4-fold) in tumours relative to normal tissues. Increased *IMPACT* expression in tumours compared to matching normal tissues indicates *IMPACT*’s important role in neoplasia.

### Association of *IMPACT* expression with gene copy number change and methylation

Intriguingly, 2.6-fold greater *IMPACT* expression was observed in Diffuse large B-cell lymphoma compared to Acute myeloid leukaemia; two haematological malignancies in TCGA (Fig. 1a). This differential expression may be explained in part by Diffuse large B-cell lymphoma having nearly 5-fold higher methylation of the *IMPACT* promoter site (median β = 0.51) compared to Acute myeloid leukaemia (median β = 0.11, Fig. 1c). Moreover, *IMPACT* expression inversely correlated to a higher degree with *IMPACT* promoter methylation in Diffuse large B-cell lymphoma (ρ = − 0.85) compared to Acute myeloid leukaemia (ρ = − 0.35; Fig. 1e). Overall, most cancer types examined have hypo-methylated *IMPACT* promoter (median β < 0.13, Fig. 1c) and their *IMPACT* expression do not substantially anti-correlate (ρ > − 0.36, Fig. 1e, Additional files 2 and 3) with *IMPACT* promoter methylation. On the other hand, in twenty-one (81%) cancer types, *IMPACT* expression positively correlated (ρ = 0.25 to 0.69; *P* < 0.05) with *IMPACT* copy number gain, consistent with *IMPACT* amplification in the majority of TCGA tumour samples (Fig. 1d, e and Additional files 2 and 3). Taken in sum, the association of *IMPACT* copy number gain with gene expression for the majority of TCGA cancer types, suggests that *IMPACT* gene amplification may be a main driver of *IMPACT* expression in human cancers.

### Association of *IMPACT* with stress response genes in normal and tumour tissues

**Fig. 2:**
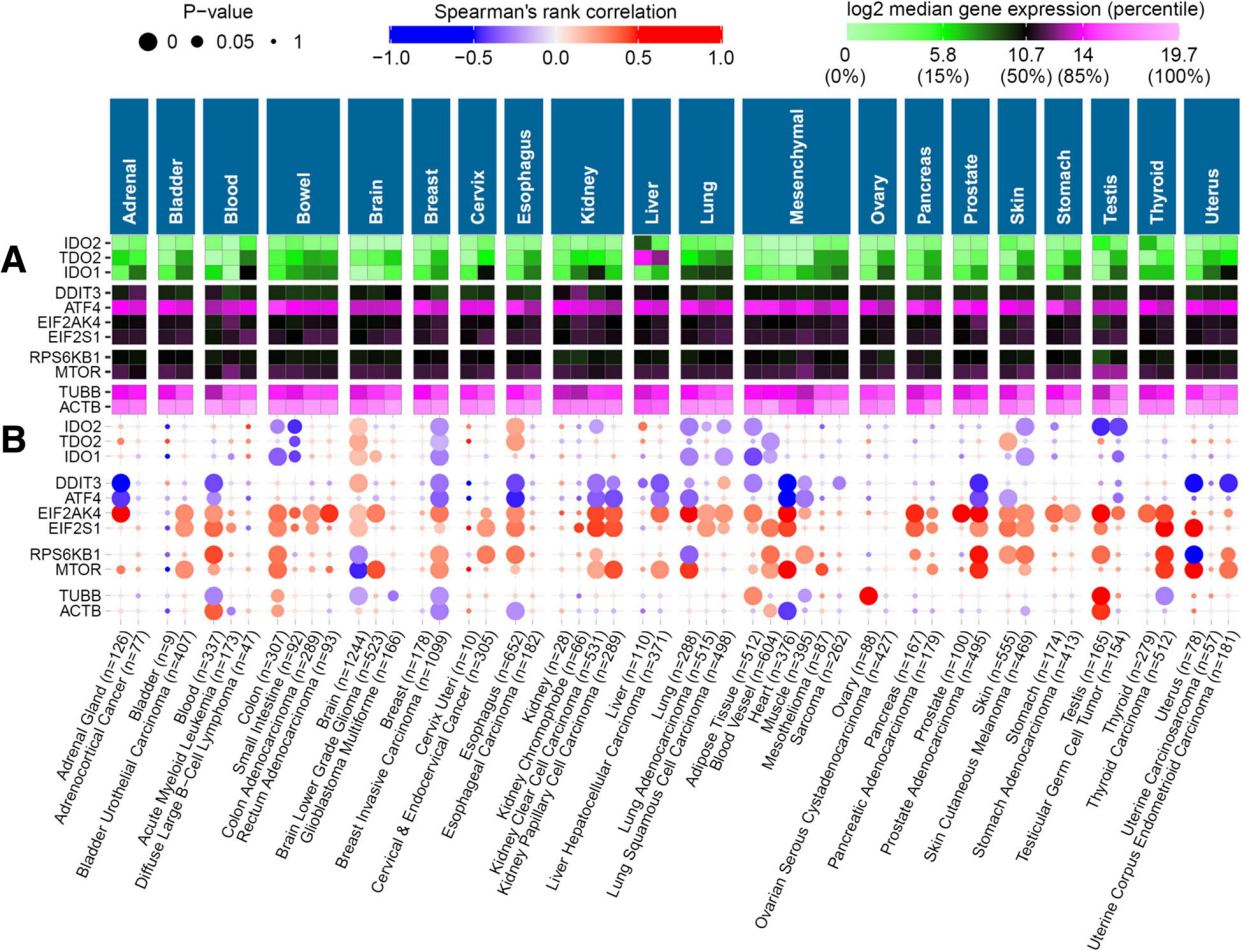
***IMPACT*** expression negatively correlates with expression of stress response genes in several clinical cancers. **a**) Spearman’s rank correlations between the expression of *IMPACT* and tryptophan dioxygenase genes (*IDO1*, *IDO2*, *TDO2*), representative genes from the GCN2 stress response pathway (*DDIT3*, *ATF4*, *EIF2AK4*, *EIF2S1*) and the mTOR pathway (*MTOR*, *RPS6KB1*), and cytoskeleton genes *ACTB* and *TUBB* as negative controls, in 28 cancer types and their 24 matching normal tissues (x-axis) classified into 20 organ categories (top panels). Colour intensity of the circles represents the magnitude of correlation and the area of circles represents statistical significance of the correlation estimated from 10^5^ random permutations adjusted for multiple comparisons using Bonferroni correction. **b**) Median mRNA counts for each gene within the dataset

We next investigated whether *IMPACT* expression levels are associated with mRNA expression signatures of stress response to tryptophan deprivation in tumours. We computed Spearman’s rank correlations between the expression of *IMPACT*, tryptophan dioxygenase genes (*IDO1*, *IDO2*, *TDO2*), and representative genes on the stress response GCN2 pathway (*EIF2AK4* (*GCN2*), *EIF2S1*, *DDIT3* (*CHOP*), *ATF4*), as well as the amino acid sensing mTOR pathway (*MTOR*, *RPS6KB1*) for all the 52 normal and cancer tissue types examined (Fig. 2). The correlations between the expression of *IMPACT* and the cytoskeleton genes encoding β-actin (*ACTB*) and β-tubulin (*TUBB*) were included as negative controls as no significant association between the expression of *IMPACT* and the two house-keeping genes was expected. Expression of *IMPACT* did not show any statistically significant positive correlations (ρ > 0.25) with the expression of *IDO1*, *TDO2* or *IDO2* in any of the 52 tissue types examined (Fig. 2a); despite *IDO1* and *TDO2* being highly expressed in the majority of the malignancies analysed (Fig. 2b). While this observation may imply that *IMPACT* expression does not associate with accelerated tryptophan catabolism in the tumour types examined, it is possible that low variance in the expression of *IMPACT* or tryptophan dioxygenase genes within each of the 52 tissue types, precludes occurrence of strong correlations. We found that the median difference between low (10th percentile) and high (90th percentile) expression of *IMPACT, IDO1*, *TDO2* and *IDO2* in the 52 tissue types examined was 2.3-fold, 26-fold, 15-fold and 10-fold, respectively. This demonstrates highly variable expression of the three tryptophan dioxygenases, but marginal variance of *IMPACT* abundance in the 52 tissue types examined which could account for the absence of strong correlations.

**Fig. 3:**
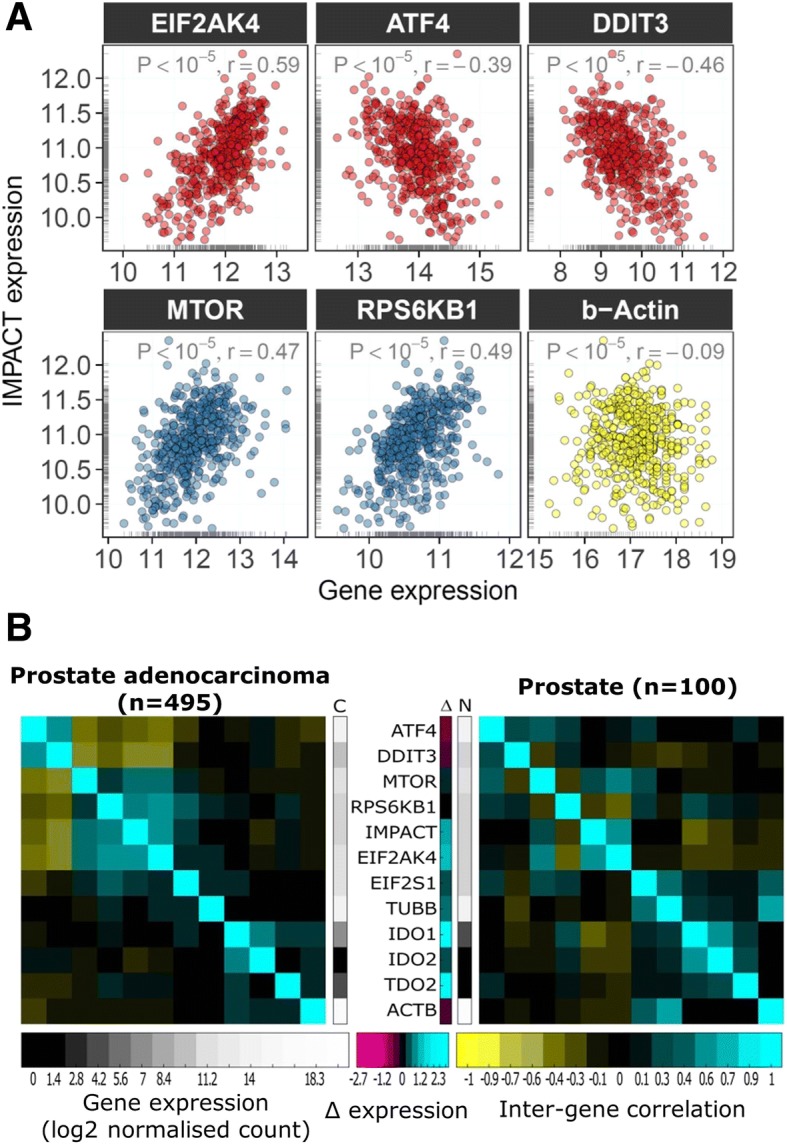
High *IMPACT* appears to ameliorate stress response when tryptophan dioxygenases are upregulated in prostate adenocarcinoma. **a**) Scatter plots between the expression of *IMPACT* (y-axis), *GCN2* pathway genes (*EIF2AK4*, *ATF4*, *DDIT3*), mTOR pathway genes (*MTOR*, *RPS6KB1*) and β-actin within the TCGA prostate adenocarcinoma cohort. P and r indicates statistical significance and correlation coefficient, respectively. Marginal grey lines represent histograms. **b**) Inter-gene correlations and differential expression of genes from Fig. 2 in prostate adenocarcinoma (left) and non-cancer prostate tissue (right). The two grey scale vertical bars denote mean gene expression value. The middle bar next to the gene names indicates differential expression (Δ) between cancer (C) and non-cancer (N) samples. The heatmap was generated using function *corplot2* from the package *pcot2* [37] in R; the Manhattan distance metric was used for clustering

It was noted that in five cancer types (breast invasive carcinoma, kidney papillary and kidney clear cell carcinomas, liver hepatocellular carcinoma and prostate adenocarcinoma), *IMPACT* expression inversely correlated (ρ = − 0.26 to − 0.46, *P* < 10^− 5^) with the expression of the central stress response transcription factor *ATF4* [29] and its downstream target, a pro-apoptotic molecule *DDIT3* (*CHOP*) [30] (Fig. 2a). In contrast, non-malignant tissue counterparts of these five cancer types do not show any statistically significant correlations (Fig. 2b) and express less *IDO1* compared to their tumour tissue counterparts (Fig. 2a). This observation is consistent with the expression of stress response genes in these five cancers being induced in response to stress caused by IDO1-dependent tryptophan catabolism. To gain further insights into the biological functions modulated by *IMPACT*, pair-wise correlations of all the genes studied were computed in the prostate adenocarcinoma cohort. Prostate adenocarcinoma was selected because it shows the strongest correlations between the expression of *IMPACT* and the other genes examined (Figs. 2b and 3a). The results of this correlation analysis demonstrate that the expression of *IMPACT*, mTOR pathway genes *RPS6KB1* and *MTOR*, and *EIF2AK4* (*GCN2*) correlate with each other, indicative of their functional relationship (Fig. 3b – left heatmap). Moreover, increased abundance of these four genes each associate with decreased expression of the stress response genes *ATF4* and *DDIT3* (Fig. 3b – left heatmap). In contrast, normal prostate tissue (Fig. 3B – right heatmap) lacks the correlation signature of its tumour counterpart indicating the absence of stress present in the respective cancer tissue. Taken together, these correlations are compatible with *IMPACT* ameliorating a stress response induced by the accelerated tryptophan catabolism in a subset of cancer types.

### Functional relationship of *IMPACT* with stress response pathways and nutrient sensing pathways

**Fig. 4:**
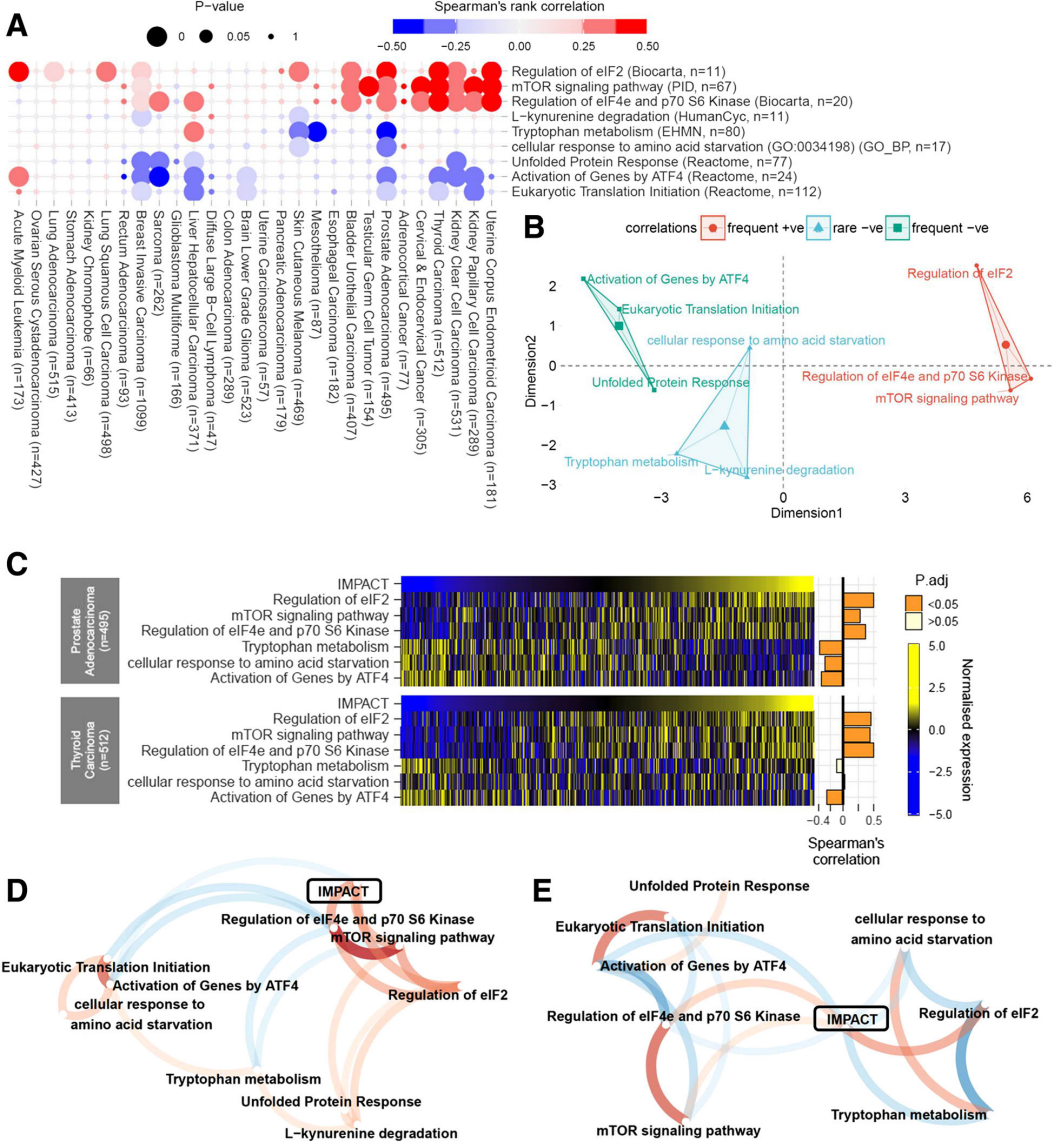
Increased *IMPACT* expression associates with elevated mTOR pathway activity and decreased stress response activity. **a**) Spearman’s rank correlations between the expression of *IMPACT* and the activity of gene sets in 28 cancer types grouped by hierarchical clustering analysis. Colour intensity and area of the circles denote magnitude and statistical significance of the correlations, respectively. **b**) Two-dimensional scaling of Manhattan distances between the gene set correlation values from panel A. Gene set proximity indicates high similarity. **c**) A heatmap of normalised gene set activities and their respective Spearman’s correlations with *IMPACT* expression (horizontal bars) in prostate (top) and thyroid (bottom) carcinoma samples. Vertical lines in the heatmap represent individual tumour samples. Network correlation plots of gene set activities and *IMPACT* expression in **d**) thyroid and **e**) prostate carcinoma. The closer the nodes, the more correlated they are. Shorter, wider and more opaque paths represent stronger correlations between the nodes and vice versa. Red and blue colours indicate positive and negative Spearman’s correlations, respectively. Paths connecting nodes correlating < |0.25| were omitted. The network correlation plots were rendered using *corrr* package [38] in R

Given the associations of *IMPACT* with genes involved in stress response and translational regulation, *IMPACT*’s functional relationship with biological pathways involving the genes analysed in Figs. 2 and 3, was subsequently examined. To this end, Spearman’s rank correlations between the expression of *IMPACT* and the relative activity of each of the 9 gene sets relevant to this hypothesis (see Methods section) were calculated for all 28 cancer types (Fig. 4a). To facilitate interpretation, two-dimensional scaling was applied to the correlation matrix on the Fig. 4a. This procedure has separated the 9 gene sets into three groups according to the frequency and magnitude of the correlations with *IMPACT* expression (Fig. 4b). The first group comprises translational regulation gene sets that positively correlate with *IMPACT* expression ((Fig. 4b) – circles). In contrast, the second group consists of gene sets associated predominantly with stress response that inversely correlate with *IMPACT* expression (Fig. 4b – rectangles). Statistically significant correlations of *IMPACT* expression with the activity of miscellaneous gene sets in the third cluster (Fig. 4b – triangles) were rarely observed and considered not significant across the cancer types examined. Although the correlation values (median ρ = 0.33 ± 0.09; Fig. 4a) were relatively modest, the correlation signature emerging from the data is consistent with *IMPACT* being an inhibitor of the GCN2-eIF2-ATF4 stress response.

Prostate and thyroid carcinomas showed the largest number of statistically significant correlations between the expression of *IMPACT* and the activity of the gene sets studied (|ρ| = 0.24 to 0.5; Fig. 4a, c). Therefore, a correlation network in these two cancer types was constructed to gain a better view on the functional relationship between the gene sets studied and *IMPACT* (Fig. 4d, e). In thyroid carcinoma (Fig. 4d), the regulation of translation initiation factor eIF2, mTOR pathway and *IMPACT* were found to be functionally related to each other. On the other hand, ATF4-mediated translation of stress response genes and cellular response to amino acid starvation, form a group which inversely correlate with the mTOR pathway and *IMPACT*. This suggests a potential involvement of *IMPACT* in restoring a normal rate of general protein synthesis through mTOR pathway and modulation of eIF2α, following attenuation of the GCN2-ATF4 stress response. A similar pattern emerges in prostate adenocarcinoma, although the clustering of *IMPACT* with the mTOR pathway is more distant compared to that in thyroid carcinoma (Fig. 4e). However, a striking association between tryptophan metabolism, *IMPACT*, regulation of eIF2 and the cellular response to amino acid starvation was noted in prostate adenocarcinoma (Fig. 4e – right). If the degree of activity of the tryptophan metabolism pathway can be used as a surrogate marker of tryptophan availability, then the data suggest that *IMPACT* expression and translational regulation increases in concert when tryptophan is in short supply (Fig. 4c, e). This would be consistent with *IMPACT* being involved in the cell’s adaptation to amino acid deficiency. However, the positive association between the activity of tryptophan metabolism and the response to amino acid starvation remains unclear (Fig. 4e).

In summary, we found concordant correlation signatures of *IMPACT* expression with genes and pathways involved in regulation of stress response and translation in a subset of cancers. This finding is consistent with *IMPACT* having a key role in conferring human cancer cells with a stronger resistance to microenvironmental stresses such as tryptophan deprivation caused by increased tryptophan dioxygenase activity.

### The role of IMPACT in cancer cell survival during tryptophan deprivation

**Fig. 5:**
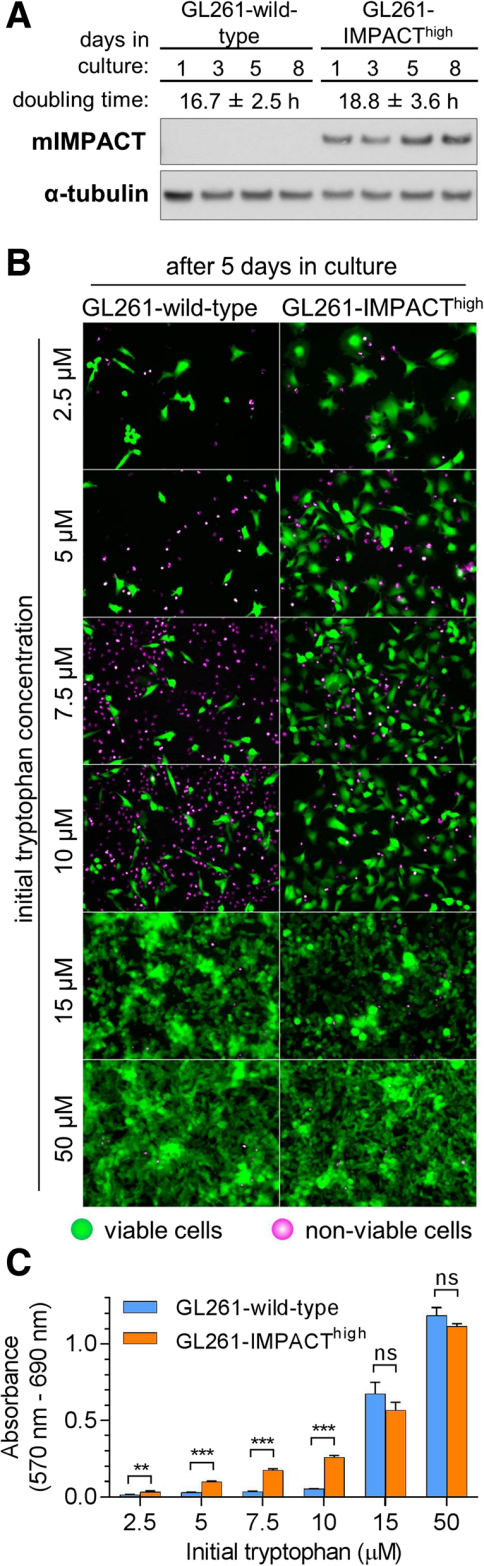
***IMPACT*** overexpression confers GL261 glioma cells increased survival during tryptophan deprivation. **a**) Western blotting with indicated antibodies on GL261-wild-type and GL261-IMPACT^high^ lines cultured in standard media containing 50 μM tryptophan over a period of 8 days. Doubling time is shown as a mean ± SD. **b**) Fluorescence microscopy images (20x objective) of GL261-wild-type and GL261-IMPACT^high^ lines stained with fluorescein diacetate and propidium iodide after five days of culture in media containing tryptophan concentrations ranging from 2.5 μM to 50 μM. **c**) Metabolic activity of GL261-wild-type and GL261-IMPACT^high^ lines determined using the MTT assay. Bar heights and whiskers represent a mean ± SD of four replicates. Statistical significance was determined using Student’s t-test in Graphpad Prism v7.03. ***P < 0.001, **P < 0.01, ns *P* > 0.25

Clinical brain tumours have one of the highest abundance of *IMPACT* transcript (Fig. 1a). We used the murine GL261 glioma line in our initial experimental studies to obtain evidence that IMPACT may be involved in enabling cancer cells to better survive periods of low tryptophan availability. The wild-type GL261 line does not express detectable levels of IMPACT protein (Fig. 5a), and these cells were engineered to constitutively express the mouse *IMPACT* gene under the control of the constitutive cytomegalovirus promoter. The engineered line that stably over-expressed IMPACT (GL261-IMPACT^high^), retained similar doubling times in culture to that of its wild-type counterpart (Fig. 5a). GL261-wild-type and GL261-IMPACT^high^ lines were subsequently cultured in low (2.5 μM to 15 μM) or high (50 μM) initial concentration of tryptophan, and viability of the cells was assessed.

After 5 days of culture in initial tryptophan concentrations ≤10 μM, cultures of IMPACT^high^ cells consistently contained a higher proportion of viable cells compared to that in cultures of wild-type cells (Fig. 5b). Differences in viability of IMPACT^high^ cells compared to wild-type, were most pronounced when cells were cultured at 7.5 μM and 10 μM initial tryptophan concentrations. Marked difference in viability between the IMPACT^high^ and wild-type cell cultures was not observed after 5 days of growth in media containing 50 μM or 15 μM tryptophan (Fig. 5b). When tryptophan is not limiting, IMPACT does not appear to exert a significant effect on GL261 cell survival. In parallel with the fluorescence microscopy study, the metabolic activity of the GL261 IMPACT^high^ and wild-type lines cultured under identical conditions, were measured using the MTT assay (Fig. 5c). The MTT data corroborated the findings from the fluorescence microscopy experiment. When cultured for 5 days at ≤10 μM initial tryptophan concentrations, the metabolic activity of both wild-type and IMPACT^high^ lines decreased by > 80% relative to that of the same cells cultured at 50 μM tryptophan. More importantly, the GL261-IMPACT^high^ cells were 4.5-fold to 7.2-fold more metabolically active compared to their wild-type counterparts (Fig. 5c). These experimental results are consistent with higher IMPACT expression conferring a greater survival advantage to cancer cells during periods of tryptophan deprivation.

**Fig. 6:**
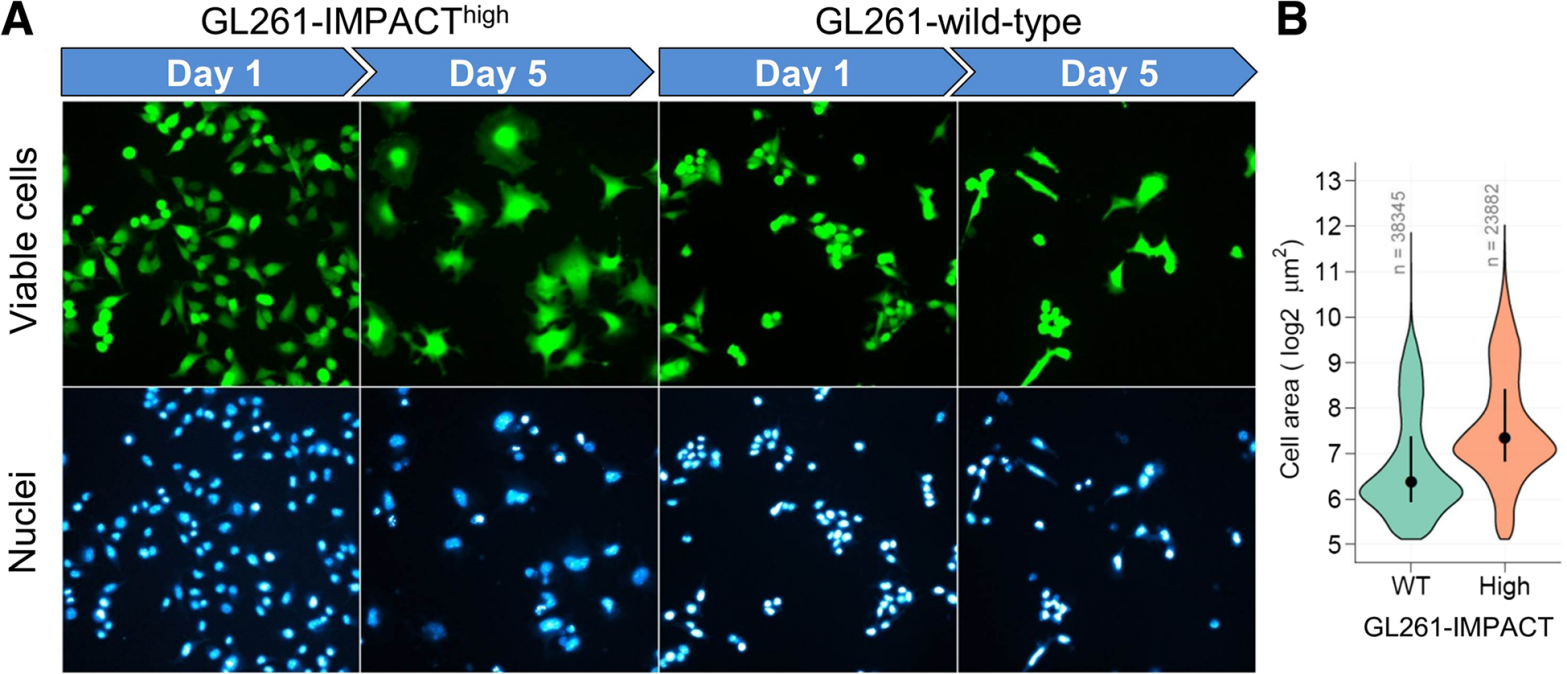
High IMPACT expression induces enlargement of tryptophan-deprived GL261 cells. **a**) Representative fluorescence images of GL261-wild-type and GL261-IMPACT^high^ cells stained with a viability marker fluorescein diacetate (green) and a DNA dye Hoechst 33342 (cyan hot) after 1 day and 5 days of culture in 2.3 μM tryptophan. **b**) Total area of viable cells after 4 days of culture in 2.3 μM tryptophan media for 4 days. Closed circles denote a median and the vertical lines represent interquartile range. n shows the number of cells analysed

As part of our studies to understand the mechanisms providing the better survival of the IMPACT^high^ cells in low tryptophan, we compared apoptosis induction in GL261-IMPACT^high^ and wild-type cells using Western blotting of cleaved PARP1 and cleaved Caspase-3. Similar extent of PARP1 cleavage was observed after 36 h and 48 h of culture of the two GL261 lines in 5 μM tryptophan. Cleaved Caspase-3 could not be detected. The better survival of IMPACT^high^ expressers did not appear to be due to a blockage of apoptotic pathways (data not shown). We noted however, that GL261-IMPACT^high^ but not GL261-wild-type cells, when cultured in the lowest tryptophan concentration studied (2.3 μM), consistently showed enlarged nuclei and cytoplasmic areas (Fig. 6a). After 5 days in culture, IMPACT^high^ cells became on average 2-fold larger than their wild-type counterparts (Fig. 6b). These enlarged cells were not observed when GL261-IMPACT^high^ or GL261-wild-type cells were cultured in the medium containing 50 μM tryptophan for 5 days. Formation of enlarged cells occurs only in IMPACT overexpressing cells and during periods of tryptophan deprivation. The appearance of these enlarged cells in culture suggests that a mechanism resembling replicative senescence may be mediating the IMPACT-dependent survival advantage to low tryptophan concentrations seen in GL261 cells.

### The relationship of IMPACT expression and the integrated stress response during periods of tryptophan deficiency

**Fig. 7:**
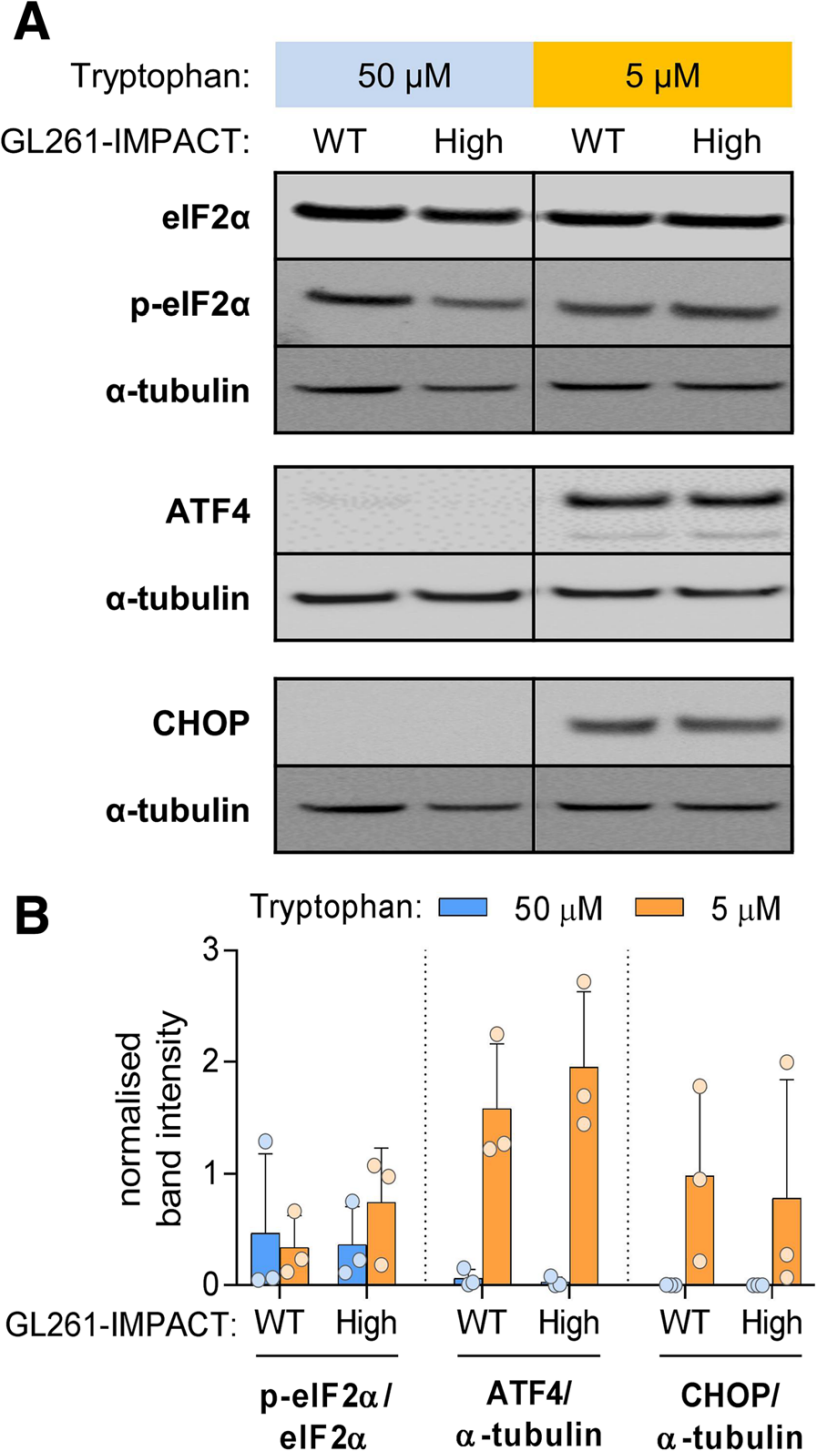
IMPACT overexpression does not appear to modulate Integrated Stress Response in tryptophan-deprived GL261 cells. **a**) A representative Western blot assessing the ISR protein content in GL261-IMPACT^high^ (High) and GL261 wild-type (WT) cells cultured for 24 h in 50 μM or 5 μM tryptophan media. **b**) Normalised band intensity of phosphorylated eIF2α, ATF4 and CHOP pooled from three independent repeats of the experiment in panel A. Bar heights and whiskers represent mean ± SD

The meta-analyses results indicate that in a subset of tumour types, high *IMPACT* expression associates with decreased expression of stress response pathways and the key ISR effector genes *ATF4* and *DDIT3 (CHOP)*. We used Western Blots to measure the expression of ATF4 and DDIT3 over the course of 24 h in GL261 cells seeded at high density in low tryptophan (5 μM) media. ATF4 and DDIT3 were detected only after 24 h of culture in 5 μM tryptophan media but not in cells cultured in 50 μM tryptophan media (Additional file 4). Phosphorylated eIF2α, ATF4 or DDIT3 in GL261 IMPACT^high^ and wild-type cells cultured for 24 h in 5 μM tryptophan were measured in three independent experiments, but no significant differences in expression were observed between the IMPACT^high^ and wild-type lines (Fig. 7a, b). The results suggested that increased survival of IMPACT^high^ GL261 cells in culture was not associated with the GCN2-dependent ISR, and other pathways are likely to be involved.

## Discussion

The data from the bioinformatics studies presented in this report support the concept that IMPACT may orchestrate an adaptive resistance mechanism in cancer cells that enables them to cope with environmental stresses; such as those elicited by amino acid deprivation induced by the immunosuppressive enzymes IDO1 and TDO2. A broad range of cancer types were found to express *IMPACT* at higher levels than that of corresponding normal tissues, and in many cases, the high expression appears to be driven by *IMPACT* amplification. However, it is not clear if *IMPACT* expression measured in the TCGA tumour samples is derived solely from cancer cells, since the majority of tumour samples would contain a mixture of cancer cells as well as stromal and immune cells. On the other hand, the meta-analysis clearly shows that whole blood samples from non-cancer donors contain the lowest levels of *IMPACT* transcript out of all non-cancer tissue types investigated. This finding indicates that human leukocytes express little to no *IMPACT,* and is in line with low expression of *IMPACT* mRNA detected in human peripheral blood leukocytes and human thymus [10], and with the low abundance of the protein in human immune T cells [12]. Given the association of *IMPACT* promoter methylation with decreased *IMPACT* expression (Fig. 1c and e), it is likely that *IMPACT* expression is epigenetically silenced in normal human blood cells, but further experiments are required to verify this. More importantly, the low abundance of *IMPACT* in human T cells provides a plausible explanation for their greater vulnerability to IDO1-mediated tryptophan catabolism [12]. In conjunction with higher *IMPACT* in cancer cells, the data from meta-analyses in this report is consistent with high IMPACT expression conferring a greater survival advantage of cancer cells over infiltrating immune T-lymphocytes that express low IMPACT, during periods of low tryptophan in the tumour microenvironment.

The GL261 cell culture results in this report are compatible with the studies in human skin fibroblasts where high endogenous expression of IMPACT renders those cells resistant to IDO1-mediated tryptophan deprivation [12, 31]. How low tryptophan concentrations reach in clinical IDO1-expressing human tumours is not well-documented, but tryptophan concentrations as low as 5 μM tryptophan, as used in our cultures, and which is sufficient to trigger a stress response in cancer cells [8], have been reported in mouse melanoma tumour models that are constitutively metabolising tryptophan [17]. Whilst *IMPACT* transcripts were detected in wild-type GL261 cells by RT-PCR (data not shown), IMPACT protein was not present, which is surprising when brains of rodents [11, 15] and primates [32] have high expression of IMPACT. Although poor correlation between gene transcript levels and protein abundance is relatively common [33], it would be of interest to determine if *IMPACT* translation is altered in cancer cells compared to that in normal cells.

The association of *IMPACT* expression with genes and pathways involved in regulation of stress response in our bioinformatics analysis (Figs. 2 and 4), while modest, is never-the-less compatible with IMPACT having a biological role as an inhibitor of the ISR in human tumours. It is also consistent with the known role of IMPACT as an inhibitor of the GCN2 stress response kinase [13, 14]. Whilst phosphorylated eIF2α, ATF4 and DDIT3 were detected in tryptophan-deprived GL261 cells (Fig. 7a, b), phosphorylated GCN2 was not consistently observed in three independent studies with GL261 glioma cells cultured in low tryptophan concentrations. The Western blots indicated that phosphorylated eIF2α and the levels of ATF4 and DDIT3 in the GL261 cell cultures was independent of GCN2 activation, and therefore one of the other three known eIF2α kinases [34] is likely to be involved. For example, tryptophan deprivation may lead to endoplasmic reticulum stress and protein misfolding that will lead to activation of the protein kinase R (PKR)-like endoplasmic reticulum kinase (PERK). Phosphorylation of eIF2α by activated PERK would compete with IMPACT/GCN2 mediated regulation of ISR.

Overall, our cell culture studies suggest that the survival advantage seen in GL261-IMPACT^high^ is mediated through a GCN2-independent pathway. Recently, IMPACT has been shown to regulate cell cycle in yeast and bind to a cyclin dependent kinase 1 (CDK1) in mammalian cells [18]; and to bind to actin in mammalian cells [19], and cleave DNA in vitro [35]. The association of IMPACT with cell cycle regulation is of particular relevance to our observations of the appearance of enlarged cells in cultures of GL261-IMPACT^high^ cells that survive tryptophan deprivation (Fig. 6a). Cell enlargement that was coupled to an increase in DNA ploidy has been linked to the development of resistance of ovarian cancer cells to the chemotherapeutic agent, paclitaxel [36]. The increase in ploidy was shown to promote DNA mutations and chromosomal rearrangements which may facilitate emergence of a more aggressive cell phenotype when the stress is relieved [36]. Further studies to delineate the mechanisms whereby IMPACT regulates cell survival and its relationship to the emergence of enlarged cells and their function in the context of survival during tryptophan deprivation are on-going in our laboratory.

## Conclusions

We provide in this report:

1. bioinformatic meta-analyses that show clinical human cancers express higher *IMPACT* compared to their normal tissue counterparts, and that *IMPACT* associates with downregulation of ATF4-mediated stress response in a subset of clinical cancers;
2. experimental data that cancer cells engineered to over-express IMPACT survive better than their wild-type counterparts when cultured under limiting tryptophan concentrations.

## Additional files

Additional file 1: Performance of 4 normalisation methods and 2 transcript abundance estimation procedures on the TOIL TCGA/GTEX dataset used in this study. **A)** Pairwise correlations of 9 normalisation/quantitation methods. Each circle represents an arithmetic mean of 30 replicated Spearman’s correlation values each calculated between 1 × 10^7^ randomly sampled gene expression pairs from each dataset (each dataset contains 3 × 10^7^ expression values; 19,446 genes × 15,741 samples). **B)** The effect of 5 normalisation techniques on differential expression (t-statistic) of 12 genes (used in Figs. 2 and 3 of the main manuscript) between each of the 28 cancer types and their respective normal tissues. t-statistic values were calculated using Welch’s unequal variance t-test. **C)** Distribution of *IMPACT, IDO1* and *TDO2* expression values across all 15,741 non-cancer (GTEX) and cancer (TCGA) samples examined in this study. **D)** Performance of 5 normalisation methods in assessing differential expression (t-statistic) of *TDO2, IDO1* and *IMPACT* in 28 cancer types relative to their corresponding normal tissues. (PDF 576 kb)

Additional file 2: Association of *IMPACT* copy number (x-axis) and a mean *IMPACT* promoter methylation (y-axis; β-value) with *IMPACT* mRNA expression in 28 different TCGA cancer types. Quantile normalised *IMPACT* mRNA expression was standardised to a mean of zero and a variance of one within each cancer type. Marginal grey lines represent histograms. Green vertical dashed lines indicate diploid gene copy number. Quantile normalised *IMPACT* expression values were standardised to a mean of 0 and a variance of 1 within each cancer type. (PDF 1021 kb)

Additional file 3: Spearman’s rank correlation between the normalised expression of IMPACT mRNA and either *IMPACT* copy number or *IMPACT* promoter methylation (median β-value) in each of the 28 TCGA cancer types studied classified into 20 tissue categories. Statistical significance of the correlations was estimated from 10^5^ random permutations and the resulting *P*-values were adjusted for multiple comparisons using a conservative Bonferroni correction. (PDF 128 kb)

Additional file 4: Expression of ATF4 and CHOP in GL261 wild-type cells cultured over the course of 24 h in low (5 μM) or high (50 μM) tryptophan media. (PDF 1546 kb)

## Abbreviations

GCN2: General control non-derepressible 2
GTEx: Genotype-Tissue Expression project
IDO1: indoleamine 2,3-dioxygenase 1
IMPACT: the product of imprinted and ancient gene
ISR: Integrated Stress Response
TCGA: The Cancer Genome Atlas
