# Supplementary material for: Imprinted and ancient gene: a potential mediator of cancer cell survival during tryptophan deprivation
Source: Cell Commun Signal. 2018 Nov 22;16:88. doi: 10.1186/s12964-018-0301-7 (PMC6251197; doi:10.1186/s12964-018-0301-7)

Transcript abundance estimation methods used: TPM = transcripts per million, RSEM = RNA-Seq by Expectation-Maximization (expected counts).

Normalisation methods used: UQ = Upper Quartile normalisation, Quant = Quantile normalisation, Rank = Gene ranking normalisation, DESeq2 = geometric mean/median ratio normalisation.

TPM and RSEM datasets, and the DESeq2-normalised RSEM dataset were downloaded from the UCSC Xena portal. UQ, Quant and Rank normalisations were applied to RSEM and TPM datasets as follows: To perform UQ normalisation, gene expression values in each sample were divided by 75th percentile of the sample's expression values. For ranking normalisation, gene expression values were ranked from lowest to highest independently within each sample; identical expression values were assigned the lowest rank in each particular group of ties. The most conservative Quantile normalisation was computed using normalize.quantiles function from a Bioconductor package preprocessCore, i.e., genes were ranked identically as in the ranking normalisation procedure but in addition, each rank was converted to an arithmetic mean of the rank's original expression values in the whole dataset to equalise statistical distribution of data between samples. DESeq2 normalisation was not applied to the TPM dataset since the DESeq2 algorithm expects raw/expected counts as an input.

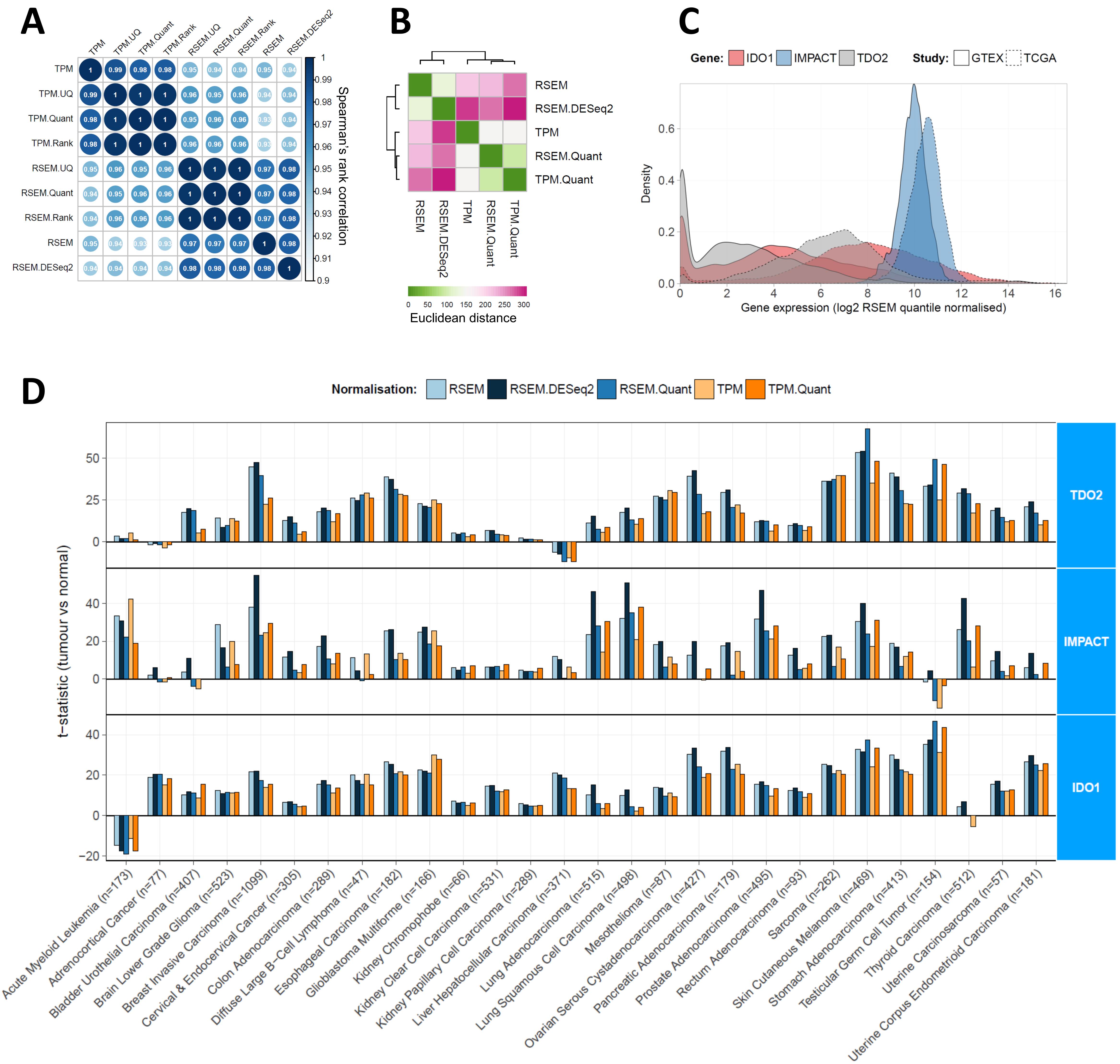

Supplement: Supplementary file 1 — Performance of 4 normalisation methods and 2 transcript abundance estimation procedures on the TOIL TCGA/GTEX dataset used in this study. A) Pairwise correlations of 9 normalisation/quantitation methods. Each circle represents an arithmetic mean of 30 replicated Spearman’s correlation values each calculated between 1 × 10^7^ randomly sampled gene expression pairs from each dataset (each dataset contains 3 × 10^7^ expression values; 19,446 genes × 15,741 samples). B) The effect of 5 normalisation techniques on differential expression (t-statistic) of 12 genes (used in Figs. 2 and 3 of the main manuscript) between each of the 28 cancer types and their respective normal tissues. t-statistic values were calculated using Welch’s unequal variance t-test. C) Distribution of IMPACT, IDO1 and TDO2 expression values across all 15,741 non-cancer (GTEX) and cancer (TCGA) samples examined in this study. D) Performance of 5 normalisation methods in assessing differential expression (t-statistic) of TDO2, IDO1 and IMPACT in 28 cancer types relative to their corresponding normal tissues. (PDF 576 kb) [file 12964_2018_301_MOESM1_ESM.pdf]
