# Supplementary figures and images for: Imprinted and ancient gene: a potential mediator of cancer cell survival during tryptophan deprivation
Source: Cell Commun Signal. 2018 Nov 22;16:88. doi: 10.1186/s12964-018-0301-7 (PMC6251197; doi:10.1186/s12964-018-0301-7)

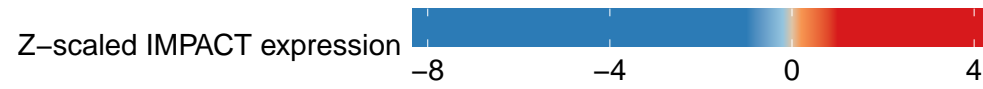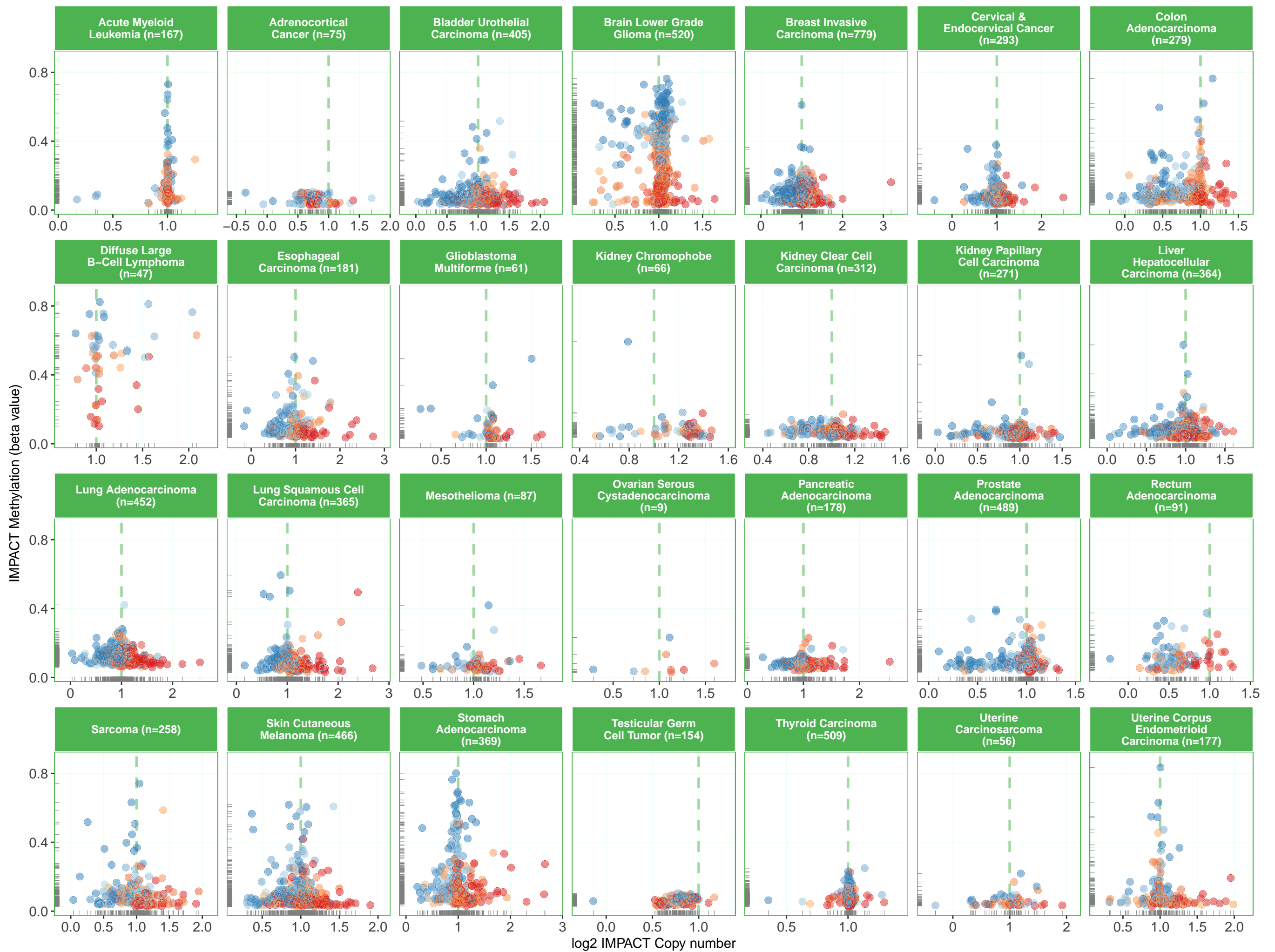

Supplement: Supplementary file 2 — Association of IMPACT copy number (x-axis) and a mean IMPACT promoter methylation (y-axis; β-value) with IMPACT mRNA expression in 28 different TCGA cancer types. Quantile normalised IMPACT mRNA expression was standardised to a mean of zero and a variance of one within each cancer type. Marginal grey lines represent histograms. Green vertical dashed lines indicate diploid gene copy number. Quantile normalised IMPACT expression values were standardised to a mean of 0 and a variance of 1 within each cancer type. (PDF 1021 kb) [file 12964_2018_301_MOESM2_ESM.pdf]
