# Supplementary material for: Imprinted and ancient gene: a potential mediator of cancer cell survival during tryptophan deprivation
Source: Cell Commun Signal. 2018 Nov 22;16:88. doi: 10.1186/s12964-018-0301-7 (PMC6251197; doi:10.1186/s12964-018-0301-7)
Supplement: Supplementary file 3 — Spearman’s rank correlation between the normalised expression of IMPACT mRNA and either IMPACT copy number or IMPACT promoter methylation (median β-value) in each of the 28 TCGA cancer types studied classified into 20 tissue categories. Statistical significance of the correlations was estimated from 105 random permutations and the resulting P-values were adjusted for multiple comparisons using a conservative Bonferroni correction. (PDF 128 kb) [file 12964_2018_301_MOESM3_ESM.pdf]

Tissue	TCGA Cancer type	# samples		Spearman's correlation		adjusted P-value	
		CNV	450K	CNV	450K	CNV	450K
Adrenal	Adrenocortical Cancer	75	77	0.33	-0.2	0.11	1
Bladder	Bladder Urothelial Carcinoma	405	407	0.51	-0.25	$<10^{-5}$	$<10^{-5}$
Blood	Acute Myeloid Leukaemia	170	170	-0.01	-0.35	1	$<10^{-5}$
	Diffuse Large B-Cell Lymphoma	47	47	-0.06	-0.85	1	$<10^{-5}$
Bowel	Colon Adenocarcinoma	287	280	0.65	-0.19	$<10^{-5}$	0.1
	Rectum Adenocarcinoma	92	92	0.55	-0.22	$<10^{-5}$	1
Brain	Brain Lower Grade Glioma	520	523	0.05	-0.7	1	$<10^{-5}$
	Glioblastoma Multiforme	160	64	0.22	-0.31	0.18	1
Breast	Breast Invasive Carcinoma	1090	785	0.46	-0.24	$<10^{-5}$	0.37
Cervix	Cervical & Endocervical Cancer	293	305	0.48	-0.28	$<10^{-5}$	$<10^{-5}$
Esophagus	Esophageal Carcinoma	181	182	0.66	-0.31	$<10^{-5}$	$<10^{-5}$
Kidney	Kidney Chromophobe	66	66	0.4	-0.05	0.03	1
	Kidney Clear Cell Carcinoma	523	317	0.37	-0.06	$<10^{-5}$	1
	Kidney Papillary Cell Carcinoma	287	273	0.41	-0.1	$<10^{-5}$	1
Liver	Liver Hepatocellular Carcinoma	364	371	0.28	-0.22	$<10^{-5}$	$<10^{-5}$
Lung	Lung Adenocarcinoma	512	455	0.55	-0.25	$<10^{-5}$	0.04
	Lung Squamous Cell Carcinoma	495	368	0.64	-0.29	$<10^{-5}$	0.56
Mesenchymal	Mesothelioma	87	87	0.38	-0.2	0.01	1
	Sarcoma	258	262	0.54	-0.32	$<10^{-5}$	$<10^{-5}$
Ovary	Ovarian Serous Cystadenocarcinoma	422	9	0.69	-0.33	$<10^{-5}$	1
Pancreas	Pancreatic Adenocarcinoma	178	179	0.59	-0.11	$<10^{-5}$	1
Prostate	Prostate Adenocarcinoma	489	495	0.37	-0.13	$<10^{-5}$	0.07
Skin	Skin Cutaneous Melanoma	466	468	0.42	-0.36	$<10^{-5}$	$<10^{-5}$
Stomach	Stomach Adenocarcinoma	412	370	0.46	-0.36	$<10^{-5}$	0.03
Testis	Testicular Germ Cell Tumor	154	154	-0.1	-0.23	1	0.11
Thyroid	Thyroid Carcinoma	509	512	0.03	-0.18	1	$<10^{-5}$
Uterus	Uterine Carcinosarcoma	56	57	0.4	-0.34	0.03	0.3
	Uterine Corpus Endometrioid Carcinoma	181	177	0.27	-0.35	0.01	$<10^{-5}$
