# Supplementary material for: Imprinted and ancient gene: a potential mediator of cancer cell survival during tryptophan deprivation
Source: Cell Commun Signal. 2018 Nov 22;16:88. doi: 10.1186/s12964-018-0301-7 (PMC6251197; doi:10.1186/s12964-018-0301-7)
Supplement: Supplementary file 4 — Expression of ATF4 and CHOP in GL261 wild-type cells cultured over the course of 24 h in low (5 μM) or high (50 μM) tryptophan media. (PDF 1546 kb) [file 12964_2018_301_MOESM4_ESM.pdf]

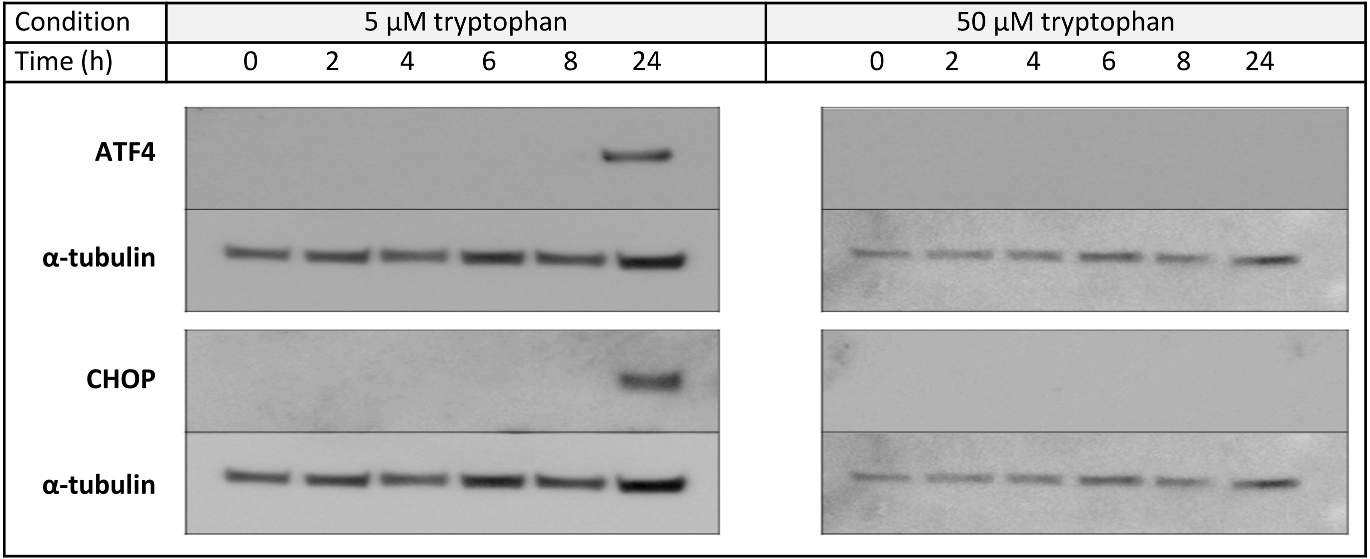
Condition
5 µM tryptophan
50 µM tryptophan
Time (h)
0
2
4
6
8
24
0
2
4
6
8
24
ATF4
α-tubulin
CHOP
α-tubulin
